# SARS-CoV-2 Variants of Concern Hijack IFITM2 for Efficient Replication in Human Lung Cells

**DOI:** 10.1128/jvi.00594-22

**Published:** 2022-05-11

**Authors:** Rayhane Nchioua, Annika Schundner, Dorota Kmiec, Caterina Prelli Bozzo, Fabian Zech, Lennart Koepke, Alexander Graf, Stefan Krebs, Helmut Blum, Manfred Frick, Konstantin M. J. Sparrer, Frank Kirchhoff

**Affiliations:** a Institute of Molecular Virology, Ulm University Medical Center, Ulm, Germany; b Institute of General Physiology, Ulm University Medical Center, Ulm, Germany; c Laboratory for Functional Genome Analysis, Gene Center, LMU München, Munich, Germany; Loyola University Chicago

**Keywords:** Omicron, SARS-CoV-2, variants of concern, iPSC-derived alveolar epithelial type II cells, interferon-induced transmembrane proteins

## Abstract

It has recently been shown that an early SARS-CoV-2 isolate (NL-02-2020) hijacks interferon-induced transmembrane proteins (IFITMs) for efficient replication in human lung cells, cardiomyocytes, and gut organoids. To date, several “variants of concern” (VOCs) showing increased infectivity and resistance to neutralization have emerged and globally replaced the early viral strains. Here, we determined whether the five current SARS-CoV-2 VOCs (Alpha, Beta, Gamma, Delta, and Omicron) maintained the dependency on IFITM proteins for efficient replication. We found that depletion of IFITM2 strongly reduces viral RNA production by all VOCs in the human epithelial lung cancer cell line Calu-3. Silencing of IFITM1 had modest effects, while knockdown of IFITM3 resulted in an intermediate phenotype. Strikingly, depletion of IFITM2 generally reduced infectious virus production by more than 4 orders of magnitude. In addition, an antibody directed against the N terminus of IFITM2 inhibited SARS-CoV-2 VOC replication in induced pluripotent stem cell (iPSC)-derived alveolar epithelial type II cells, thought to represent major viral target cells in the lung. In conclusion, endogenously expressed IFITM proteins (especially IFITM2) are critical cofactors for efficient replication of genuine SARS-CoV-2 VOCs, including the currently dominant Omicron variant.

**IMPORTANCE** Recent data indicate that SARS-CoV-2 requires endogenously expressed IFITM proteins for efficient infection. However, the results were obtained with an early SARS-CoV-2 isolate. Thus, it remained to be determined whether IFITMs are also important cofactors for infection of emerging SARS-CoV-2 VOCs that outcompeted the original strains in the meantime. This includes the Omicron VOC, which currently dominates the pandemic. Here, we show that depletion of endogenous IFITM2 expression almost entirely prevents productive infection of Alpha, Beta, Gamma, Delta, and Omicron SARS-CoV-2 VOCs in human lung cells. In addition, an antibody targeting the N terminus of IFITM2 inhibited SARS-CoV-2 VOC replication in iPSC-derived alveolar epithelial type II cells. Our results show that SARS-CoV-2 VOCs, including the currently dominant Omicron variant, are strongly dependent on IFITM2 for efficient replication, suggesting a key proviral role of IFITMs in viral transmission and pathogenicity.

## INTRODUCTION

Since its first occurrence in Wuhan, China, in December 2019, the severe acute respiratory syndrome coronavirus 2 (SARS-CoV-2), the causative agent of coronavirus disease 2019 (COVID-19), has caused a devastating pandemic (1, 2). The reasons for the efficient spread of this coronavirus are not fully understood but clearly involve the ability to efficiently infect and propagate in human cells. Viral entry depends on binding of the viral Spike (S) protein to the cellular angiotensin-converting enzyme 2 (ACE2) receptor and proteolytic processing of the S precursor into the active S1 and S2 subunits. However, additional host factors may affect the efficiency of SARS-CoV-2 entry and play roles in viral transmission and pathogenesis (3).

We recently demonstrated that interferon (IFN)-inducible transmembrane proteins (IFITM1, -2, and -3) are required for efficient SARS-CoV-2 infection (4). This came as surprise since IFITMs are a family of IFN-stimulated genes (ISGs) that are well known to protect cells against infection by numerous viral pathogens, including retro-, flavi-, influenza-, rhabdo-, filo-, and bunyaviruses (5–7). Inhibitory effects have also been reported for highly pathogenic coronaviruses, including SARS-CoV-2 (8, 9). However, most evidence was obtained using pseudoparticles containing the S protein of SARS or Middle East respiratory syndrome (MERS) coronaviruses, cells that are not intrinsically permissive to this virus and/or artificially overexpress IFITM proteins. Notably, it has been reported that the common cold coronavirus OC43 hijacks IFITM3 for efficient entry (10).

The antiviral mechanism of IFITMs is thought to involve alterations in the rigidity and curvature of the cellular membrane, affecting viruses in a broad, unspecific way (6, 7, 11). In contrast, results of coimmunoprecipitation and proximity ligation assays as well as inhibition studies support that the SARS-CoV-2-enhancing effect involves specific interactions between the S protein and the N-terminal region of IFITMs, especially IFITM2, promoting subsequent virus-cell fusion in early endosomes (4). In accordance with hijacking of IFITMs for efficient SARS-CoV-2 infection, knockdown (KD) of endogenous IFITM2 expression in human lung cells strongly reduced viral infection and infectious virus production. In addition, IFITM2-derived peptides as well as an IFITM2-targeting antibody protected gut organoids and cardiomyocytes against infection and cytopathic effects of SARS-CoV-2 (4).

In the initial study, IFITM dependency for efficient infection has only been demonstrated for an early European variant of SARS-CoV-2 isolated in the Netherlands in February 2020 (NL-02-2020) (4). Since then, numerous variants have emerged. Some of them show increased transmission fitness and immune evasion and are thus referred to as “variants of concern” (VOCs). Currently, the WHO has categorized five SARS-CoV-2 variants as VOCs: B.1.1.7, B.1.351, P.1, B.1.617.2, and B.1.1.529. The first four, referred to as Alpha, Beta, Gamma, and Delta variants, have previously spread in the human population, and the fifth (Omicron) is currently dominating the pandemic. Compared to the NL-02-2020 isolate, all VOCs contain various alterations in their S proteins reported to enhance viral infectivity, transmissibility, and pathogenicity by affecting ACE2 receptor affinity, proteolytic activation, and susceptibility to neutralization (12–16). This applies in particular to the Omicron VOC, which contains more than 30 amino acid changes in the S protein compared to the early Wuhan strain, many of them located in the binding domain for the ACE2 receptor (16–18). Since its first identification in November 2021 in South Africa, it has outcompeted the previously dominating Delta VOC at amazing speed (https://nextstrain.org/ncov/gisaid/global). Compelling evidence shows that the Omicron VOC shows increased transmission efficiency and escape from neutralizing antibodies compared to other SARS-CoV-2 VOCs (16, 19–22). Changes in the S protein might affect IFITM interaction and dependency both directly and indirectly: for example, by altering the affinity for the primary ACE2 receptor or the dependency on specific proteases and hence the primary site of fusion between the viral and cellular membranes. Here, we examined whether current SARS-CoV-2 VOCs, including Omicron, still require IFITM proteins for efficient replication in human lung cells.

## RESULTS

**FIG 1:**
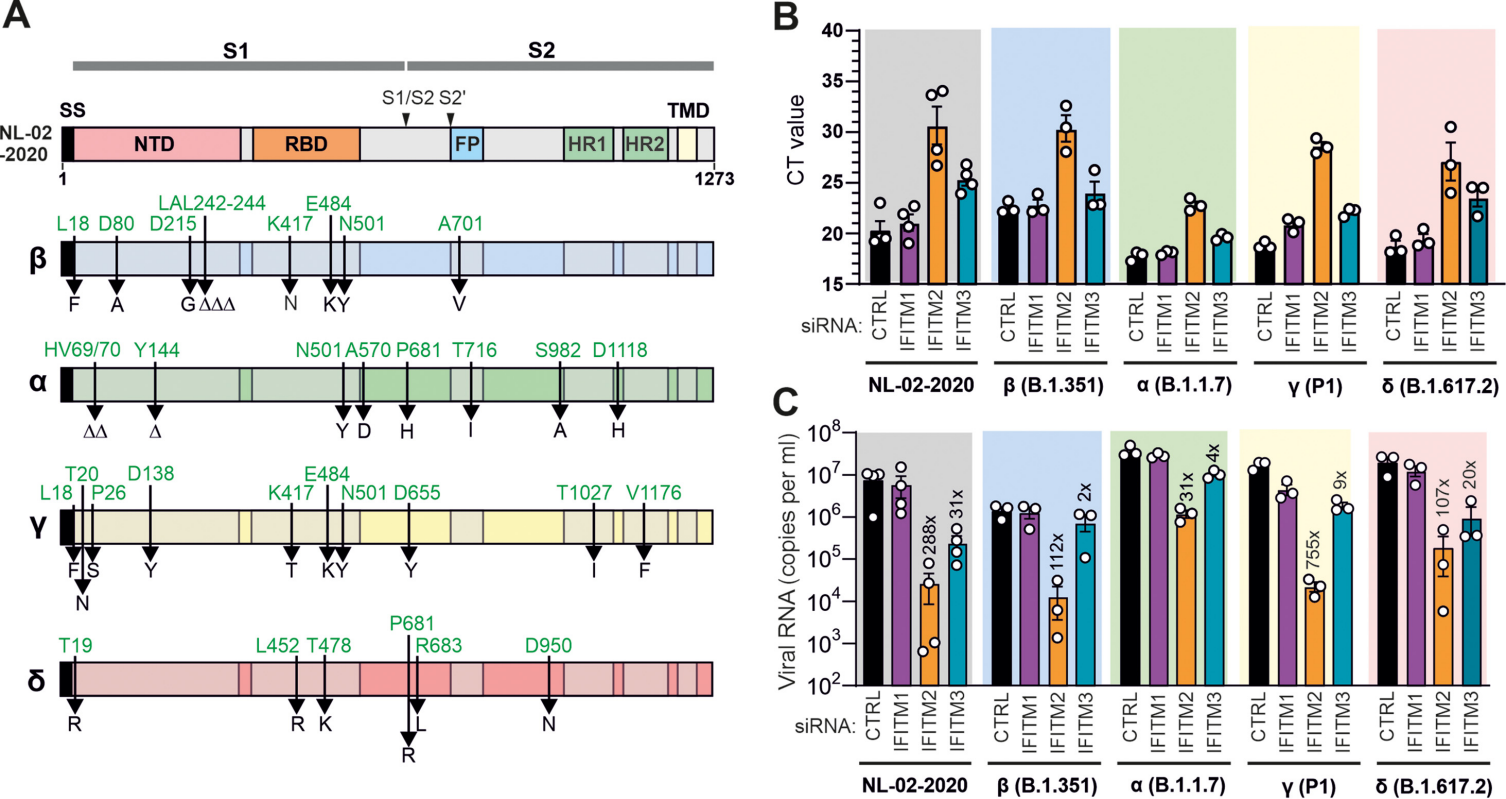
Role of IFITMs in replication of SARS-CoV-2 VOCs in Calu-3 cells. (A) Amino acid variations in the Spike proteins of SARS-CoV-2 Alpha, Beta, Gamma, and Delta variants were investigated. The upper panel shows a schematic representation of the SARS-CoV-2 S protein with specific domains indicated in different colors. Abbreviations: SS, signal sequence; NTD, N-terminal domain; RBD, receptor binding domain; FP, fusion peptide; HR, heptad repeat; TMD, transmembrane domain. The S1/S2 and S2′ proteolytic cleavage sites are also indicated. (B) Raw qRT-PCR *C_T_* values obtained using supernatants of Calu-3 cells collected 2 days postinfection; (C) viral N RNA levels in the supernatant of Calu-3 cells infected with the indicated SARS-CoV-2 variants (MOI of 0.05). Cells were transfected with control (CTRL) or IFITM-targeting siRNAs as indicated. Numbers above the bars indicate *n*-fold reduction compared to the viral RNA levels detected in the supernatant of Calu-3 cells treated with control siRNA. Bars in panels A and B represent the mean from 3 to 4 independent experiments (±standard error of the mean [SEM]), each measured in technical duplicates.

To verify that the SARS-CoV-2 VOCs show the expected differences in S compared to the early NL-02-2020 isolate, we performed full-genome sequence analyses of the four variants initially available for functional analyses. The NL-02-2020 isolate already contains the D614G amino acid substitution, which has been reported to increase SARS-CoV-2 transmissibility (23) and is also found in all current VOCs (24, 25). As expected, the Spike proteins of the four initial VOCs differed by 6 to 10 amino acid changes from the NL-02-2020 Spike (Fig. 1A). The Alpha VOC (B.1.1.7) that emerged at the end of 2020 in the United Kingdom contains eight mutations in its S protein: deletions of H69/V70 and Y144 and mutations of N501Y, A570D, P681H, T716I, S982A, and D1118H (26). The Beta VOC (B.1.351) emerged in South Africa in October 2020 and has initially spread to all continents (27). Its S protein contains three alterations in the receptor binding domain (RBD) (K417N, E484K, N501Y) and five additional changes (L18F, D80A, D215G, R246I, A701V). The Gamma (P.1) variant was first detected in Brazil at the end of 2020 and shares the K417T, E484K, and N501Y S mutations with the Alpha and/or Beta VOC (Fig. 1A) (28). The Delta (B.1.617.2) variant was first identified in India in the first half of 2021 (29) and temporarily outcompeted all other SARS-CoV-2 VOCs around the globe. It differs by changes of T19R, deletion of residues 157 and 158, and L452R, T478K, E484K, P681R, R683L, and D950N from NL-02-2020 in the S protein (Fig. 1A). Several changes (L18F, K417T, E484K, and N501Y) emerged independently by convergent evolution in several VOCs (13, 30). The reasons why they are associated with a selective advantage remain to be fully elucidated, but rapidly accumulating evidence supports that they reduce neutralization by antibodies and/or increase ACE2 binding affinity (13). In addition, the P681R substitution near the furin cleavage site might improve proteolytic activation of the Delta S protein (31–34). Thus, all four VOCs contain changes in their S proteins reported to increase interaction with their primary ACE2 receptor. A stronger affinity for the primary ACE2 receptor may reduce viral dependency on other entry cofactors required for efficient entry and fusion, such as IFITM proteins.

To examine the role of endogenous IFITM expression on infection by genuine SARS-CoV-2 VOCs, we performed small interfering RNA (siRNA) knockdown (KD) studies in the human epithelial lung cancer cell line Calu-3, which endogenously express ACE2 and all three IFITM proteins (4). Viral replication was determined by quantification of viral N (nucleocapsid) RNA levels by quantitative reverse transcription-PCR (qRT-PCR) in the cell culture supernatants 2 days after infection with the five SARS-CoV-2 variants (Fig. 1B). The Alpha, Gamma, and Delta VOCs produced 2- to 4-fold-higher levels of viral RNA (vRNA) than NL-02-2020 in Calu-3 cells, while the Beta variant showed moderately reduced levels of viral RNA production (Fig. 1C). Silencing of IFITM2 expression reduced viral RNA production from 31-fold (Alpha) to 754-fold (Gamma). Replication of the Beta variant was 112-fold reduced in the absence of IFITM2. In comparison, KD of IFITM1 had little effect, while silencing of IFITM3 resulted in a minimum of 2-fold (Beta) to a maximum of 31-fold (NL-02-2020) lower viral RNA yields (Fig. 1C). IFITM2 KD reduced viral RNA yields by the Delta variant by >100-fold, while IFITM3 silencing was associated with a 20-fold reduction. Altogether, the results confirmed our previous findings for the NL-02-2020 strain (4) and further demonstrated that the four VOCs still require IFITMs for efficient infection.

**FIG 2:**
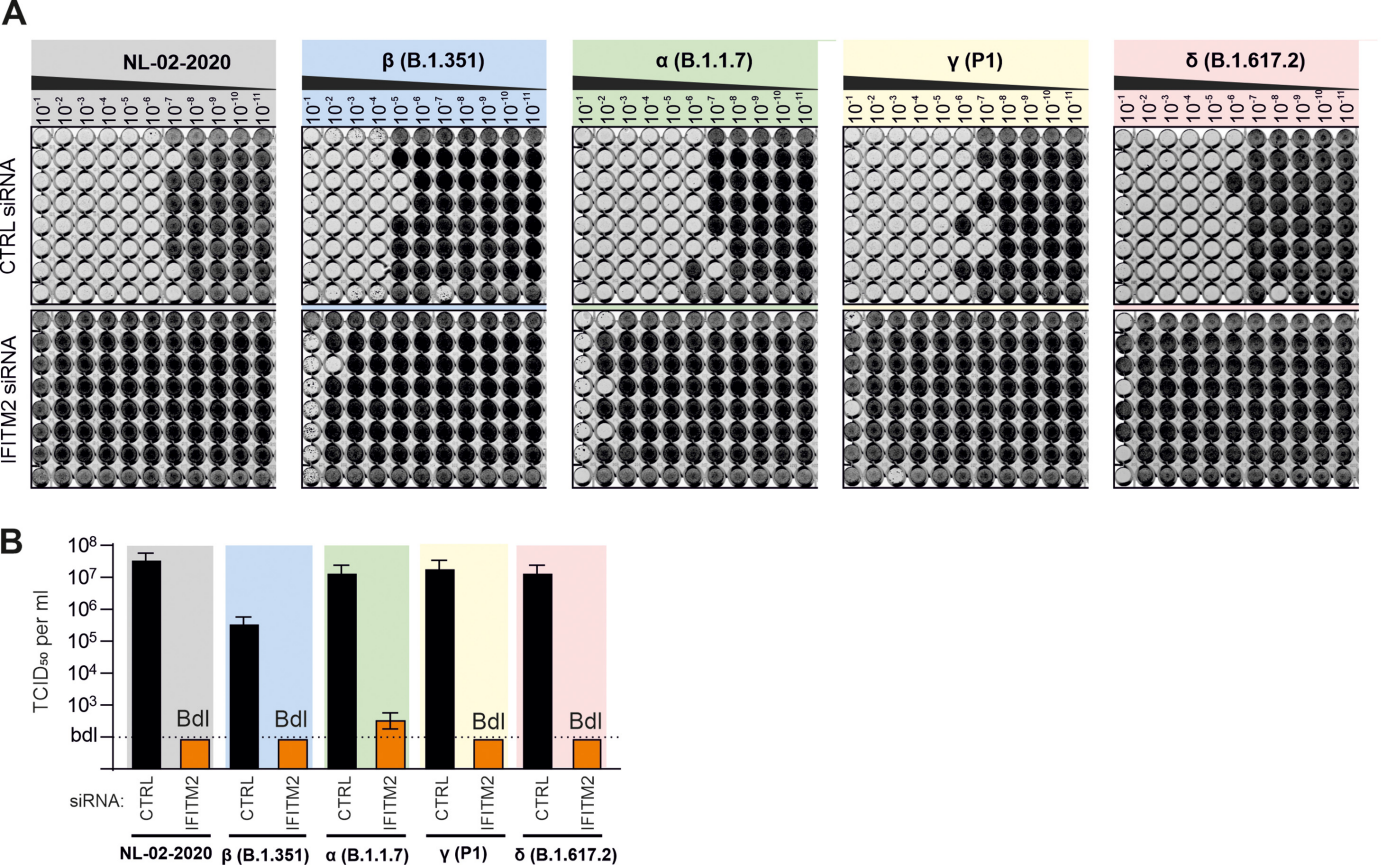
Silencing of endogenous IFITM2 expression prevents production of infectious SARS-CoV-2. (A) Supernatants derived from Calu-3 cells treated with control or IFITM2 siRNA 2 days after infection with the SARS-CoV-2 NL-02-2020 or the indicated VOCs were serially diluted and added to Caco-2 cells seeded into 96-well plates. Five days later, cells were examined for CPE, fixed, and stained with crystal violet. Productively infected wells appear transparent since the cells are eliminated or detached due to viral infection. (B) Quantification of infectious SARS-CoV-2 particles in the supernatant of Calu-3 cells treated with control or IFITM2 targeting siRNAs. Bars represent the mean of one experiment performed with eight technical replicates (±standard deviation [SD]) shown in panel A. Bdl, below detection limit.

To further determine whether IFITM2 is critical for productive replication of these SARS-CoV-2 VOCs in Calu-3 cells, we determined the 50% tissue culture infectious dose (TCID_50_) of viral particles in the cell culture supernatants (Fig. 2A). With the exception of the Beta variant, which showed the lowest viral RNA yields (Fig. 1C) and infectious titers, all SARS-CoV-2 variants produced more than 10 million infectious virus particles/mL culture supernatant in Calu-3 cells treated with the control siRNA (Fig. 2B). In striking contract, infectious virus in the supernatant was generally reduced by 4 to 6 orders of magnitude, to levels near or below background (≤100 infectious particles/mL) upon silencing of IFITM2 (Fig. 2). Altogether, these results show that the SARS-CoV-2 Alpha, Beta, Gamma, and Delta VOCs are all strongly dependent on endogenous IFITM2 expression for efficient replication in Calu-3 lung cells.

**FIG 3:**
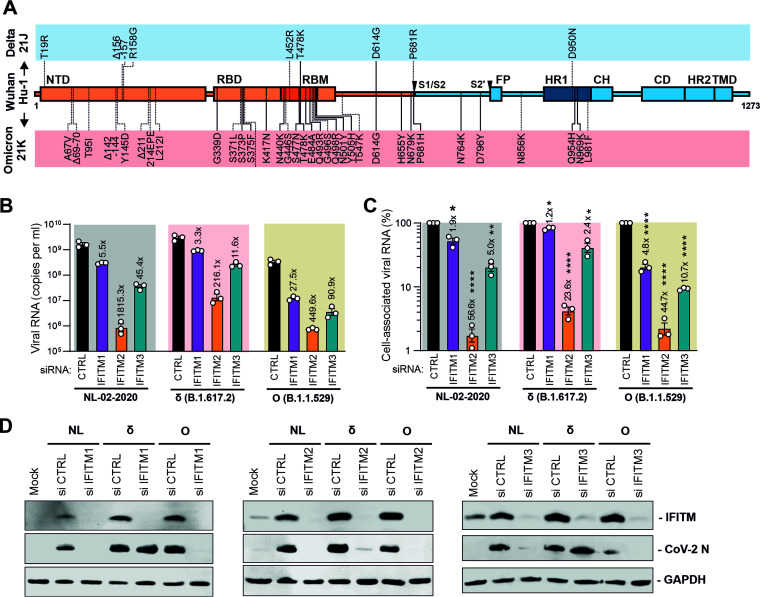
Impact of IFITMs on replication of the Omicron VOC. (A) Schematic presentation of amino acid variations in Spike proteins of the SARS-CoV-2 Delta and Omicron VOCs investigated. Abbreviations are defined in the legend to Fig. 1A. (B) Viral N RNA levels in the supernatant of Calu-3 cells infected with the indicated SARS-CoV-2 variants. Cells were transfected with control or IFITM-targeting siRNAs as indicated. Numbers above the bars indicate *n*-fold reduction compared to the viral RNA levels detected in the supernatant of Calu-3 cells treated with control siRNA. Bars in panels B and C show the mean of 3 independent experiments (±SEM), each measured in technical duplicates. (C) Quantification of intracellular viral N RNA levels in Calu-3 cells 24 h postinfection with SARS-CoV-2 (MOI of 0.05). Values were normalized to GAPDH and calculated relative to the control (set to 100%). Cells were transiently transfected with siRNA—either control (CTRL) or targeting IFITM1, -2, or -3. (D) Exemplary Western blot of IFITM and viral N protein expression in Calu-3 cells treated with control or IFITM1-, -2-, and/or -3-targeting siRNA and infected with the indicated SARS-CoV-2 variants. GAPDH was detected as a loading control. *P* values: *, <0.05; **, <0.01; ****, <0.0001.

Our study was initiated prior to the discovery of the Omicron VOC therefore, this variant was analyzed in an independent set of experiments. Sequence analysis confirmed the presence of numerous amino acid changes and several deletions in the Spike protein compared to the Wuhan Hu-1 strains and the Delta VOC (Fig. 3A). In agreement with the results obtained using the remaining SARS-CoV-2 variants, silencing of IFITM2 had the strongest effect of the three IFITM proteins and reduced viral RNA production of the Omicron VOC by ~450-fold (Fig. 3B). Notably, replication of the Omicron variants was also significantly affected by knockdown of IFITM1 and IFITM3, which reduced viral RNA production by about 28- and 90-fold, respectively (Fig. 3B). Similar effects were observed on the levels of cell-associated viral RNA (Fig. 3C), and the reduction of cell-free and cell-associated viral RNA levels upon knockdown of the three IFITM proteins correlated significantly (*R*^2^ = 0.8854; *P* < 0.001; *n* = 12). Western blot analyses confirmed efficient knockdown of the IFITM proteins by the respective siRNAs and revealed reduced expression of the viral N proteins, especially upon silencing of IFITM2 (Fig. 3D). In agreement with the effects on viral RNA production, silencing of IFITM1 and IFITM3 had the least effect on the Delta VOC. Most importantly, the results show that the dominant Omicron VOCs remained strongly dependent on IFITMs for efficient replication in human lung cells.

**FIG 4:**
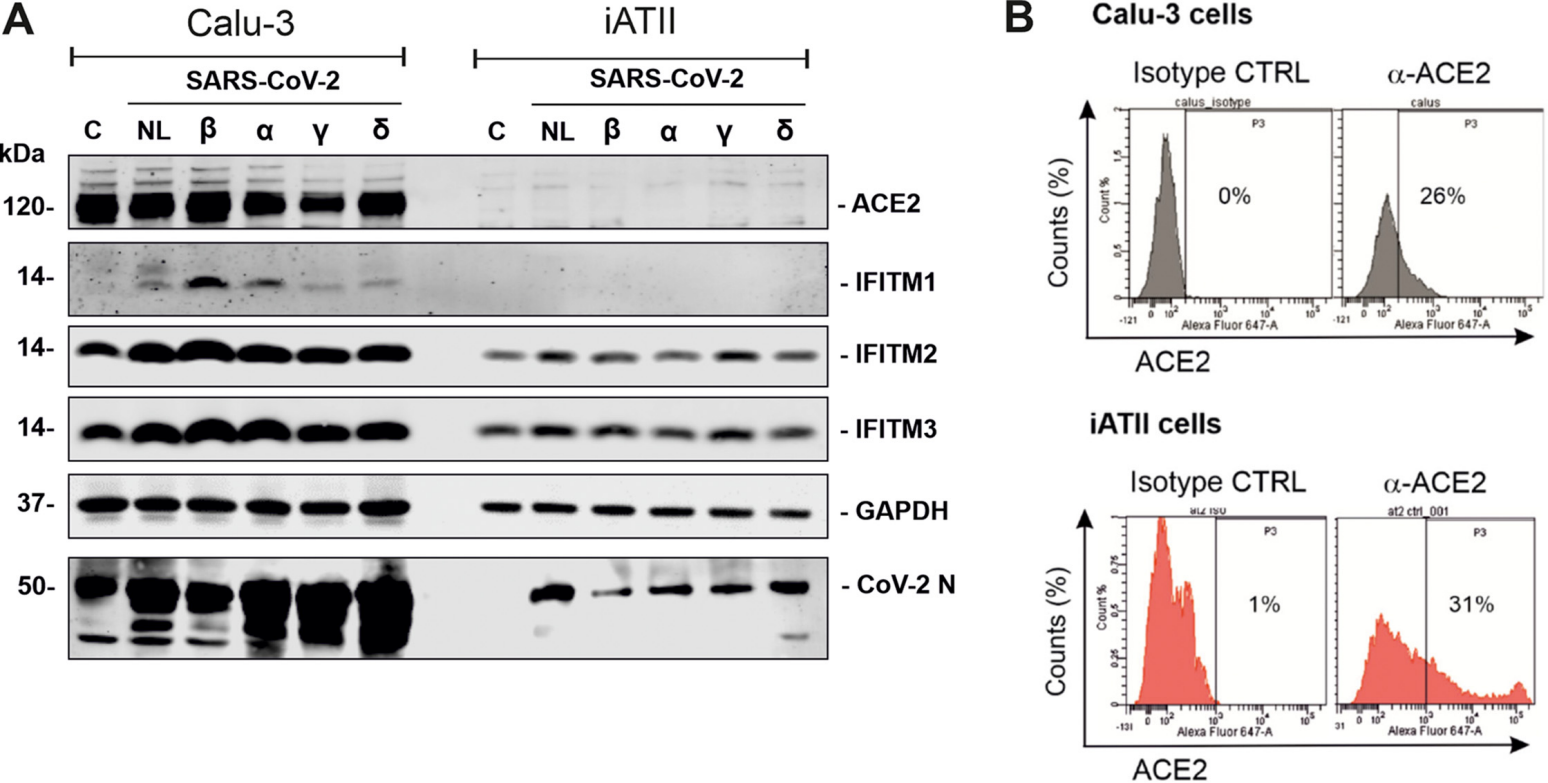
Expression of ACE2 and IFITM proteins in Calu-3 and iATII cells. (A) Immunoblot of ACE2, IFITM1, IFITM2, and IFITM3 in Calu-3 and iATII cells left uninfected (lanes C) or infected with the indicated SARS-CoV-2 variants. Whole-cell lysates were stained with the indicated antibodies. An unspecific signal was observed in the Calu-3 control lane stained with the CoV-2 N antibody. (B) Flow cytometric analysis of surface ACE2 expression in Calu-3 and iATII cells.

The structure of IFITM proteins has not been determined, and their membrane topology is under debate and may even vary, depending on the cell type (3, 35). We have previously shown that an antibody targeting the N terminus of IFITM2 inhibits replication of the NL-02-2020 isolate in gut organoids and cardiomyocytes (4). To further examine the potential relevance of IFITM2 for transmission of SARS-CoV-2 VOCs, we performed experiments in induced pluripotent stem cell (iPSC)-derived alveolar epithelial type II (iATII) cells as a model for the main target cells of SARS-CoV-2 infection in the distal lung (36). Western blot analyses showed that, similarly to Calu-3 cells, iATII cells express IFITM2 and IFITM3 (Fig. 4A). In contrast, both cell types showed little (Calu-3) or no (iATII) detectable expression of IFITM1. Unexpectedly, we detected only marginal levels of ACE2 expression in iATII cells by Western blot analyses, while ACE2 was readily detectable in Calu-3 cells (Fig. 4A). In agreement with previous data (37); however, ACE2 expression by iATII cells was clearly detectable by flow cytometry (Fig. 4B). The reasons for this discrepancy remain to be determined, but it is tempting to speculate that a high proportion of ACE2 is located at the surface of iATII cells.

**FIG 5:**
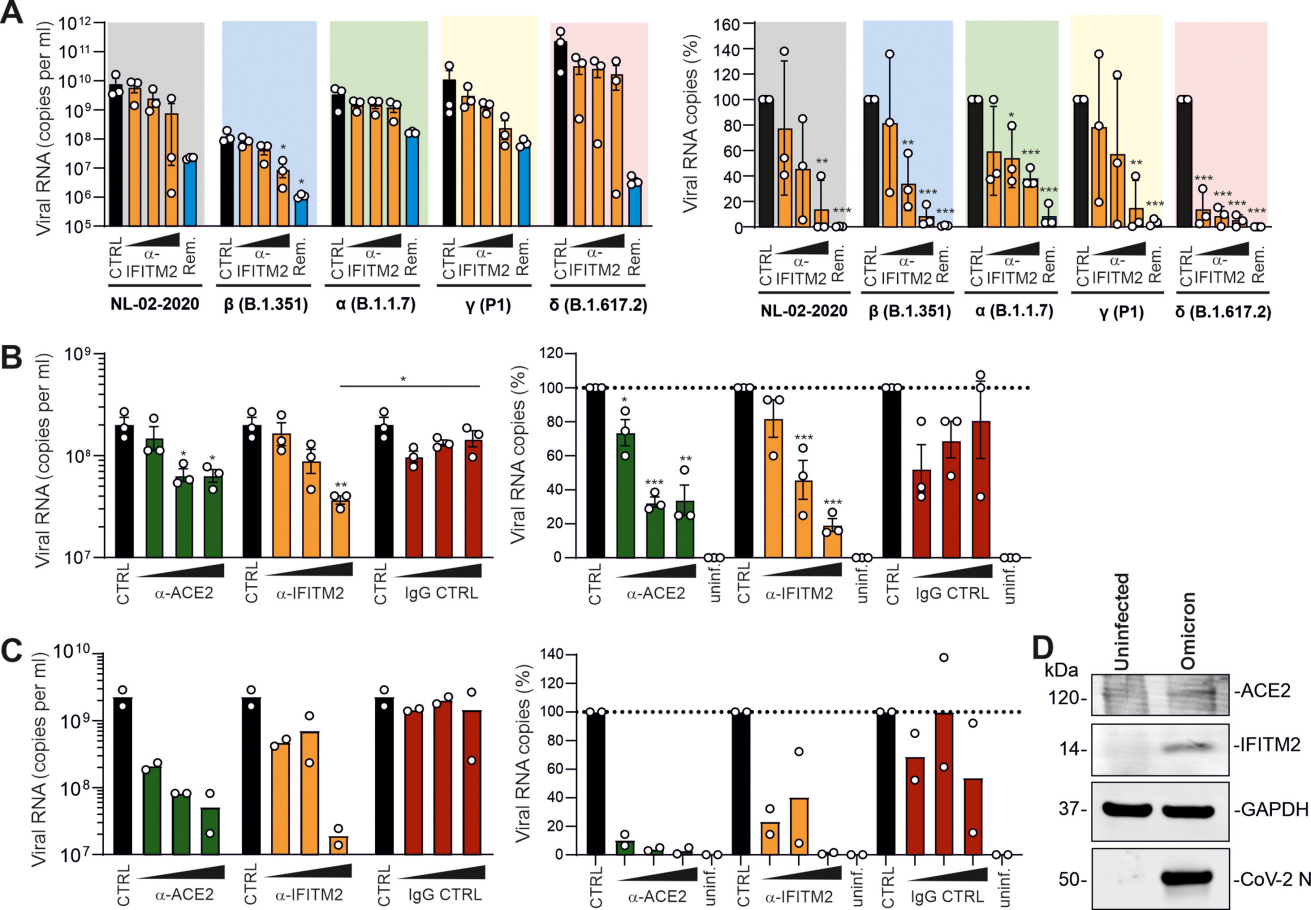
Effect of an anti-IFITM2 antibody on replication of SARS-CoV-2 variants in iATII cells. (A) Quantification of viral N RNA levels in the supernatant of iATII cells treated with anti-IFITM2 antibody (20, 40, or 80 μg/mL) or remdesivir (10 μM) 1 h before infection (SARS-CoV-2, MOI of 0.5), collected 48 h postinfection. Bars represent the mean from three independent experiments. The right panel shows the reduction of vRNA levels in the supernatants relative to the untreated control (100%). *P* values: *, <0.05; **, <0.01; ***, <0.001. (B, C) Quantification of viral N RNA levels in the supernatant of iATII cells collected at 48 h postinfection with SARS-CoV-2 at an MOI of 0.5 and pretreated (1 h before infection) with increasing concentrations (20, 40, or 80 μg/mL) of the indicated antibodies (anti-IFITM2, anti-IgG, and anti-ACE2) or remdesivir (10 μM). Cells were infected with the NL-02-2020 (B) or Omicron B.1.1.529 (C) variants. Bars represent the mean from three (B) or two (C) independent experiments. (D) Immunoblot of ACE2 and IFITM2 in iATII cells left uninfected or infected with the SARS-CoV-2 Omicron variant. Whole-cell lysates were stained with the indicated antibodies.

In agreement with published data (37–39), iATII cells were highly susceptible to SARS-CoV-2 replication (Fig. 1C and Fig. 5A). On average, the Delta variant replicated to about 30-fold-higher levels (average vRNA copy numbers of 2.4 × 10^11^) than the early NL-02-2020 isolate in iATII cells (Fig. 5A, left). The broad-spectrum antiviral agent remdesivir (40) efficiently inhibited replication of all SARS-CoV-2 variants. Treatment of iATII cells with the antibody against the N terminus of IFITM2 also generally reduced viral RNA production in a dose-dependent manner, albeit with various levels of efficiency (Fig. 5A, right). In agreement to our previous study, treatment with antibodies directed against ACE2 or the N-proximal region of IFITM2 inhibited infection by the NL-02-2020 strain, while an isotype control antibody had no significant inhibitory effect (Fig. 5B). Similarly, the Omicron VOC was susceptible to inhibition by both ACE2 and IFITM2 antibodies, and almost complete inhibition was observed at the highest doses (Fig. 5C). It has been previously shown that innate immune activation and virus infection may induce ACE2 and IFITM2 expression (4, 41). Western blot analyses revealed increased expression levels of both cellular factors in Omicron-infected compared to uninfected iATII cells (Fig. 5D). Altogether, the results agree with our previous finding that IFITM2 can be targeted to protect various types of human cells against SARS-CoV-2 infection (4).

## DISCUSSION

In the present study, we demonstrate that IFITMs (especially IFITM2) are also critical cofactors for efficient replication of all five SARS-CoV-2 VOCs, including the currently dominant Omicron variant. We have previously shown that IFITMs promote SARS-CoV-2 replication in primary small airway epithelial cells (SAECs) and that IFITM2 can be targeted to inhibit viral replication in gut organoids and cardiomyocytes derived from human iPSCs (4). The present finding that an anti-IFITM2 antibody inhibits replication of SARS-CoV-2 VOCs in iPSC-derived alveolar epithelial type II cells, proposed to model the main target cells of SARS-CoV-2 infection in the distal lung (37, 39), adds to the evidence that IFITM2 might play a key role in SARS-CoV-2 transmission, dissemination, and pathogenesis. Our observation that IFITM2 dependency is maintained by VOCs also further underlines that, against the odds, this cellular “antiviral-turned-proviral” factor represents a potential target for therapeutic or preventive approaches in COVID-19.

All five current SARS-CoV-2 VOCs required IFITMs (especially IFITM2) for efficient replication. However, there are some notable differences. For example, the Alpha variant yielded ~100-fold-higher levels of vRNA upon silencing of IFITM2 expression than the 2019 CoV-2 and Beta variants (Fig. 1C). Thus, it is tempting to speculate that this VOC did not only evolve reduced susceptibility to IFN inhibition (42, 43) but may also be less dependent on IFITM2 for efficient infection than other SARS-CoV-2 variants. While efficient replication of all VOCs was most strongly dependent on IFITM2, silencing of IFITM1 and IFITM3 impaired replication of the Omicron VOC more severely than that of the remaining SARS-CoV-2 VOCs. The reasons for this remain to be determined, but it is tempting to speculate that alterations in the efficiency and preferential site of viral fusion may contribute to differences between Omicron and other VOCs. For example, several studies reported that Omicron entry may have shifted toward the TMPRSS2-independent endosomal route (22, 44, 45). It has been reported that IFITM1 localizes mainly to the plasma membrane, while IFITM2 and IFITM3 localize preferentially to early and late endosomes, respectively (5). Thus, alterations in preferred sites of fusion may shift viral dependency on specific IFITM proteins.

In agreement with previous findings (31, 42), the Delta variant replicated with higher efficiency than the early SARS-CoV-2 isolate in human lung cells, particularly in iPSC-derived alveolar epithelial type II cells (Fig. 5A). This agrees with recent data showing that the Delta variant infects human bronchial epithelial cells with higher efficiency than other VOCs (46). In comparison, it has been reported that the Omicron VOC replicates less efficiently than the Delta VOC in TMPRSS2-expressing cells (45). This agrees with our finding that the levels of viral RNA production achieved by the Delta variant in ATII cells, which express high levels of TMPRSS2 (38, 47), were substantially higher than those obtained for the Omicron VOC (Fig. 5). Similarly, the Omicron VOC produced lower levels of viral RNA than the NL-02-2020 isolate and the Delta VOC in Calu-3 cells (Fig. 3) that endogenously express TMPRSS2 (48), although the difference was smaller than in ATII cells. These findings add to the evidence that the Spike protein of the Omicron VOC is not generally more active than that of other VOCs (45) but may spread most efficiently because it efficiently evades neutralizing antibodies and shows alterations in cell tropism (49).

Our results are only seemingly at odds with studies reporting that IFITMs inhibit SARS-CoV-2 (8, 50, 51). There is agreement that overexpression of IFITM proteins efficiently inhibits Spike-containing pseudovirions and genuine SARS-CoV-2 (4, 8). However, our findings that endogenous IFITM expression is critical for efficient replication of genuine SARS-CoV-2 in human lung and gut cells (4) are not contradicted by experimental evidence since inhibitory effects of IFITMs were generally observed under more artificial conditions. Notably, our results are in line with the recent identification of IFITM proteins as SARS-CoV-2 dependency factors in Vero E6 cells (52). It has been previously shown that IFITMs promote infection by human coronavirus OC43 (10), and recent data suggest that IFITM1 and -3 promote infection by herpesviruses and hepatitis B and D viruses (53, 54). Thus, a variety of viral pathogens might be able to utilize IFITMs to their advantage.

Our discovery of IFITMs as important cofactors of SARS-CoV-2 infection is not contradicted by the finding that IFITM3 knockout increases viral pathogenicity in mice (51). Rodent and human IFITMs share only ~60% homology. Viral entry involves direct protein-protein interactions at multiple interfaces, and even small changes at these contact sites can affect tropism. Indeed, a much higher level of homology between murine and human ACE2 (82%) is insufficient to facilitate efficient infection of regular mice with SARS-CoV-2. The ability of SARS-CoV-2 to hijack IFITM proteins (especially IFITM2) for efficient infection seems to require specific interactions between the N-terminal portion of IFITMs and the viral Spike protein (4). In contrast, the inhibitory effect of IFITM proteins is broad and involves changes in cellular membrane rigidity and curvature instead of specific interactions with viral glycoproteins (55–57). While further studies are required to fully elucidate the mechanisms underlying inhibitory and enhancing effects of IFITMs, it is conceivable that the broad inhibitory effect is species independent, while promotion of viral entry may represent a species-specific adaptation of the SARS-CoV-2 Spike protein.

The exact topology of IFITM proteins remains to be defined. Different models have been proposed, and IFITMs might even exist in different conformations (7, 35). In agreement with previous data on IFITM3 (35), we found that the N-terminal part of IFITM2 is accessible for inhibitory antibodies at the cell surface. While IFITM2 and IFITM3 share high levels of amino acid identity, the region targeted by the inhibitory antibody that might represent the Spike interaction site varies substantially between both IFITM proteins (4). Together, with their differential subcellular localization (5, 6) these sequence variations may explain why IFITM2 is more critical for SARS-CoV-2 infection than IFITM3.

The Alpha, Delta, and Omicron variants contain a mutation of P681H/R close to the furin cleavage site that might affect interferon sensitivity, proteolytic activation, and fusogenicity of the S protein (33, 34, 58). The Alpha and Delta VOCs produced the highest levels of viral RNA upon IFITM2 KD. In comparison, the Omicron variant generally produced lower levels of viral RNA in Calu-3 cells than the Delta variant and was more strongly affected by KD of IFITM1 and IFITM2 than the remaining VOCs. Thus, it will be interesting to further determine whether an altered intrinsic fusogenic activity and TMPRSS2 dependency of SARS-CoV-2 VOCs affect their dependency on specific IFITM proteins for efficient infection. Our previous results clearly support that IFITM2 promotes SARS-CoV-2 entry into its target cells (4). It is noteworthy, however, that IFITM2 KD generally had stronger effects on infectious viral titers than on viral RNA yields. Thus, it will be of interest to further clarify whether the presence of IFITM2 indeed has an enhancing effect on the infectiousness of SARS-CoV-2 particles or if the background levels are just higher for viral RNA due to release from or lysis of infected cells.

Importantly, the dominant Omicron variant, which contains a striking number of about 30 amino acid changes in the Spike protein, many of which are located in the ACE2 binding site compared to the Wuhan strain (14, 59), remains strongly dependent on IFITMs for efficient infection. Altogether, our results add to the evidence that IFITMs are critical for efficient SARS-CoV-2 replication and support that IFITM2 remains an unexpected but well-suitable target for therapeutic approaches against this pandemic viral pathogen, including its emerging variants.

## MATERIALS AND METHODS

### Cell culture.

Calu-3 cells (a human epithelial lung adenocarcinoma line) were cultured in Eagle’s minimum essential medium (MEM) (Sigma, catalog no. M4655) supplemented with 10% (upon and after viral infection) or 20% (during all other times) heat-inactivated fetal bovine serum (FBS) (Gibco, catalog no. 10270106), 100 U/mL penicillin, 100 μg/mL streptomycin (Thermo Fisher, catalog no. 15140122), 1 mM sodium pyruvate (Pan Biotech, catalog no. P04-8010), and 1× nonessential amino acids (Sigma, catalog no. M7145). Vero E6 cells (a Cercopithecus aethiops-derived epithelial kidney line; ATCC) and TMPRSS2-expressing Vero E6 cells (kindly provided by the National Institute for Biological Standards and Control [NIBSC], no. 100978) were grown in Dulbecco’s modified Eagle’s medium (DMEM) (Gibco, catalog no. 41965039) supplemented with 2.5% (upon and after viral infection) or 10% (during all other times) heat-inactivated FBS (Gibco, catalog no. 10270106), 100 U/mL penicillin, 100 μg/mL streptomycin (Thermo Fisher, catalog no. 15140122), 2 mM l-glutamine (Gibco, catalog no. 25030081), 1 mM sodium pyruvate (Pan Biotech, catalog no. P04-8010), 1× nonessential amino acids (Sigma, catalog no. M7145), and 1 mg/mL Geneticin (Gibco, catalog no. 10131-019) (for TMPRSS2-expressing Vero E6 cells). Caco-2 cells (a human epithelial colorectal adenocarcinoma line, kindly provided by Holger Barth (Ulm University)) were grown in the same medium as Vero E6 cells, but with supplementation with 10% heat-inactivated FBS.

Human induced alveolar type 2 (iATII) cells were differentiated from BU3 NKX2-1^GFP^;SFTPC^tdTomato^-induced pluripotent stem cells (iPCSs) (60) (kindly provided by Darrell Kotton, Boston University and Boston Medical Center) and maintained as alveolospheres embedded in three-dimensional (3D) Matrigel in CK+DCI medium, as previously described (61). For infection studies, iATII cells were cultured as 2D cultures on Matrigel-coated plates in CK+DCI medium plus 10 μM Y-27632 (Tocris, catalog no. 1254) for 48 h before switching to CK+DCI medium on day 3.

### SARS-CoV-2 stocks.

The SARS-CoV-2 variant B.1.351 (Beta), 2102-cov-IM-r1-164, was provided by Michael Schindler (University of Tübingen), and the B.1.617.2 (Delta) variant was provided by Florian Schmidt (University of Bonn). The BetaCoV/Netherlands/01/NL/2020 (NL-02-2020), B.1.1.7. (Alpha), and hCoV-19/Netherlands/NH-EMC-1720/2021, lineage B.1.1.529 (Omicron), variants were obtained from the European Virus Archive. The hCoV-19/Japan/TY7-503/2021 (Brazil P.1) (Gamma) (NR-54982) isolate was obtained from the BEI resources. SARS-CoV-2 strains were propagated on Vero E6 (NL-02-2020, Delta), Vero E6 overexpressing TMPRSS2 (Alpha), CaCo-2 (Beta), or Calu-3 (Gamma, Omicron) cells. To this end, 70 to 90% confluent cells in 75-cm^2^ cell culture flasks were inoculated with the SARS-CoV-2 isolate (multiplicity of infection [MOI] of 0.03 to 0.1) in 3.5 mL serum-free medium. The cells were incubated for 2 h at 37°C, before adding 20 mL medium containing 15 mM HEPES (Carl Roth, catalog no. 6763.1). Virus stocks were harvested as soon as strong cytopathic effect (CPE) became apparent. The virus stocks were centrifuged for 5 min at 1,000 × *g* to remove cellular debris, aliquoted, and stored at −80°C until further use.

### Sequencing of SARS-CoV-2 VOCs.

Virus stocks were inactivated and lysed by adding 0.3 mL TRIzol reagent (Ambion, catalog no. 132903) to 0.1 mL virus stock. Viral RNA was isolated using the Direct-zol RNA miniprep kit (ZymoResearch, catalog no. R2050) according to the manufacturer’s instructions, eluting the RNA in 50 μL DNase/RNase-free water. The protocol to prepare the viral RNA for sequencing was modified from the nCoV-2019 sequencing protocol V.1. For reverse transcription, the SuperScript IV (SSIV) first-strand synthesis system (Invitrogen, catalog no. 18091050) was used with modified manufacturer’s instructions. First, 1 μL random hexamers (50 ng/μL), 1 μL deoxynucleoside triphosphate (dNTP) mix (10 mM each), and 11 μL template RNA (diluted 1:10 in DNase/RNase free water) were mixed, and then the mixture was incubated at 65°C for 5 min and placed on ice for 1 min. Next, 4 μL SSIV buffer, 1 μL dithiothreitol (DTT; 100 mM), 1 μL RNaseOUT RNase inhibitor, and 1 μL SSIV reverse transcriptase were added to the mixture, followed by incubation at 24°C for 5 min, 42°C for 50 min, and 70°C for 10 min. To generate 400-nucleotide (nt) fragments in PCR, the ARTIC nCoV-2019 V3 primer set (IDT) and the Q5 Hot Start high-fidelity 2× master mix (NEB, catalog no. M0494S) were used with modified manufacturer’s instructions. The primer pools 1 and 2 were diluted to a final concentration of 10 μM, and a reaction with each primer pool was set up as follows: 4 μL respective primer pool, 12.5 μL Q5 Hot Start high-fidelity 2× master mix, 6 μL water, and 2.5 μL cDNA. The PCR was performed as follows: 98°C for 30 s, followed by 30 cycles of 98°C for 15 s and 65°C for 5 min, and hold at 4°C. The PCR products were run on a 1% agarose gel to check for the presence of fragments at the correct size. The products from primer pool 1 and primer pool 2 for each variant were pooled, diluted, and quantified by Qubit DNA HS kit (Thermo Fisher, catalog no. Q32851). The sequencing amplicon pools were diluted to 0.2 ng/μL and tagmented with Nextera XT library prep kit (Illumina, catalog no. FC-131-1024). Nextera libraries were dual barcoded and sequenced on an Illumina NextSeq1000 instrument. The obtained sequenced reads were demultiplexed and mapped against the SARS-CoV-2 reference genome (GenBank accession no. NC_045512.2) with *BWA-MEM* (62). Pileup files were generated from the mapped reads using *Samtools* (63). The mapped reads and the pileup file were used to construct the consensus sequence with the *iVar* package (64) using default settings.

### IFITM knockdown.

At 24 h and 96 h postseeding, 150,000 Calu-3 cells, seeded into 24-well plates, were transfected with 20 μM nontargeting siRNA or IFITM1-, IFITM2-, or IFITM3-specific siRNAs (IFITM1 siRNA pool, GGACAGCACCAAA, CCCAAGGCCACCGGA, GACAGAAAACAGGAA, and CCCAGACCAGGAC; IFITM2 siRNA pool, CAAACCCCCCGCA, GGCGGCCCGCAA, CAAGGAGGAGCAGAAG, and CGCCAGGCCCAGCGAA; IFITM3 siRNA pool, ACGGCGGGCAAA, CGCACCCAGGCGA, GCGACCCAGGCCA, and AGGAAGACAGGAGGCA) (Dharmacon, Horizon) using Lipofectamine RNAiMAX (Thermo Fisher, catalog no. 13778100) according to the manufacturer’s instructions. Six hours after the second transfection, Calu-3 cells were infected with the various SARS-CoV-2 variants at an MOI of 0.05. Six hours postinfection, the inoculum was removed, and the cells were washed once with PBS and supplemented with fresh media. At 48 h postinfection, supernatants were harvested for quantitative reverse transcription-PCR (qRT-PCR) analysis.

### qRT-PCR.

N (nucleoprotein) transcript levels were determined in supernatants collected from SARS-CoV-2-infected Calu-3 cells 48 h postinfection as previously described (65). Total RNA was isolated using a viral RNA minikit (Qiagen, catalog no. 52906) according to the manufacturer’s instructions. RT-qPCR was performed as previously described (66) using TaqMan Fast Virus 1-Step master mix (Thermo Fisher, catalog no. 4444436) and a OneStepPlus real-time PCR system (96-well format, fast mode). Primers were purchased from Biomers (Ulm, Germany) and dissolved in RNase-free water. Synthetic SARS-CoV-2-RNA (Twist Bioscience, catalog no. 102024) or RNA isolated from BetaCoV/France/IDF0372/2020 viral stocks quantified via this synthetic RNA (for low-threshold-cycle [*C_T_*] samples) were used as a quantitative standard to obtain viral copy numbers. All reactions were run in duplicates. The primer and probe sequences were as follows: forward primer (HKU-NF), 5′-TAATCAGACAAGGAACTGATTA-3′; reverse primer (HKU-NR), 5′-CGAAGGTGTGACTTCCATG-3′; probe (HKU-NP), 5′-6-carboxyfluorescein (FAM)-GCAAATTGTGCAATTTGCGG-6-carboxytetramethylrhodamine (TAMRA)-3′.

### Inhibition assays.

A total of 30,000 iATII cells were seeded as single cells into 96-well plates coated for 1 h at 37°C with 0.16 mg/mL Matrigel (Corning, catalog no. 356238) diluted in DMEM/F-12 (Thermo Fisher, catalog no. 11330032). Twenty-four hours later, cells were treated with increasing concentrations (20, 40, 80 μg/mL) of anti-IFITM2 (Cell Signaling, catalog no. 13530 S), anti-ACE2 AK (AC18Z) (Santa Cruz Biotechnology, catalog no. sc-73668), or normal rabbit anti-IgG (Cell Signaling, catalog no. 2729) or remdesivir (10 μM) (Selleck Chemicals catalog no. S8932). At 1 h post-treatment, cells were infected with SARS-CoV-2 VOCs at an MOI of 0.5. At 6 h postinfection, cells were washed once with PBS and supplemented with fresh medium. Thereafter, the day 0 wash control was harvested. Forty-eight hours postinfection, supernatants were harvested for qRT-PCR analysis.

### Western blot.

To determine the expression of cellular and viral proteins, infected Calu-3 (MOI of 0.2, 48 h postinfection) or iATII (MOI of 0.5, 48 h postinfection) cells or uninfected controls were washed in PBS and subsequently lysed in Western blot lysis buffer (150 mM NaCl, 50 mM HEPES, 5 mM EDTA, 0.1% NP-40, 500 μM Na_3_VO_4_, 500 μM NaF [pH 7.5]) supplemented with protease inhibitor cocktail (Roche, catalog no. 11697498001). After 5 min of incubation on ice, samples were centrifuged (4°C, 20 min, 20,817 × *g*) to remove cell debris. The supernatant was transferred to a fresh tube, and the protein concentration was measured by Nanodrop and adjusted using Western blot lysis buffer. Western blotting was performed as previously reported. Proteins were stained at 1:1,000 using primary antibodies against IFITM1 (Cell Signaling, catalog no. 13126S), IFITM2 (Cell Signaling, catalog no. 13530 S), IFITM3 (Cell Signaling, catalog no. 59212S), ACE2 (rabbit polyclonal) (Abcam, catalog no. ab166755), rat anti-GAPDH (Biolegend, catalog no. 607902), and SARS CoV-2 N (Sino Biologicals, catalog no. 40588-V08B) and infrared dye-labeled secondary antibodies (Li-Cor IRDye). Membranes were scanned using an Odyssey infrared imager.

### TCID_50_ endpoint titration.

A total of 10,000 Caco-2 cells were seeded into 96-well F-bottom plates. One day later, infectious supernatants were serially diluted and added to the cells. Cells were then incubated for 5 days and monitored for CPE. Cells were fixed with 4% paraformaldehyde (PFA) at room temperature for 30 min. After the cells were washed with PBS once, 100 μL of staining solution (0.5% crystal violet and 0.1% Triton in water) was added. After 20 min of incubation at room temperature, the staining solution was removed using water, and the TCID_50_ per milliliter was calculated according to the method of Reed and Muench (67).

### Flow cytometric analysis.

A total of 60,000 iATII cells or Calu-3 cells were incubated for 1 h at 4°C with equal protein concentrations of control rabbit IgG (Diagenode, catalog no. C15410206) or rabbit anti-ACE2 (Abcam, catalog no. ab166755) diluted in FACS buffer (PBS, 1% FBS), washed three times in PBS, stained for 30 min with a 1:400 dilution of goat anti-rabbit AF647 (Invitrogen, catalog no. A27040), fixed in 1% PFA, and analyzed using a BD FACS Canto II flow cytometer.

### Statistical analysis.

Statistical analysis was performed using GraphPad Prism software. Two-tailed unpaired Student's *t* test was used to determine statistical significance. Significant differences are indicated as follows: *, *P* < 0.05; **, *P* < 0.01; ***, *P* < 0.001; **** *P* < 0.0001. Statistical parameters are specified in the figure legends.
